# The efficacy of the modified Atkins diet in North Sea Progressive Myoclonus Epilepsy: an observational prospective open-label study

**DOI:** 10.1186/s13023-017-0595-3

**Published:** 2017-03-07

**Authors:** Martje E. van Egmond, Amerins Weijenberg, Margreet E. van Rijn, Jan Willem J. Elting, Jeannette M. Gelauff, Rodi Zutt, Deborah A. Sival, Roald A. Lambrechts, Marina A. J. Tijssen, Oebele F. Brouwer, Tom J. de Koning

**Affiliations:** 1 Department of Neurology, University Medical Centre Groningen, University of Groningen, PO Box 30.001, 9700 RB Groningen, The Netherlands; 2 Department of Neurology, Ommelander Ziekenhuis Groningen, PO Box 30.000, 9670 RA Winschoten, The Netherlands; 3 Department of Paediatrics, University Medical Centre Groningen, University of Groningen, PO Box 30.001, 9700 RB Groningen, The Netherlands; 4 Department of Genetics, University Medical Centre Groningen, University of Groningen, PO Box 30.001, 9700 RB Groningen, The Netherlands

**Keywords:** Myoclonus, Epilepsy, Treatment, Ketogenic diet, Modified Atkins diet, *GOSR2* gene, Quality of life, North Sea Progressive Myoclonus Epilepsy

## Abstract

**Background:**

North Sea Progressive Myoclonus Epilepsy is a rare and severe disorder caused by mutations in the *GOSR2* gene. It is clinically characterized by progressive myoclonus, seizures, early-onset ataxia and areflexia. As in other progressive myoclonus epilepsies, the efficacy of antiepileptic drugs is disappointingly limited in North Sea Progressive Myoclonus Epilepsy. The ketogenic diet and the less restrictive modified Atkins diet have been proven to be effective in other drug-resistant epilepsy syndromes, including those with myoclonic seizures. Our aim was to evaluate the efficacy of the modified Atkins diet in patients with North Sea Progressive Myoclonus Epilepsy.

**Results:**

Four North Sea Progressive Myoclonus Epilepsy patients (aged 7–20 years) participated in an observational, prospective, open-label study on the efficacy of the modified Atkins diet. Several clinical parameters were assessed at baseline and again after participants had been on the diet for 3 months. The primary outcome measure was health-related quality of life, with seizure frequency and blinded rated myoclonus severity as secondary outcome measures.

Ketosis was achieved within 2 weeks and all patients completed the 3 months on the modified Atkins diet. The diet was well tolerated by all four patients. Health-related quality of life improved considerably in one patient and showed sustained improvement during long-term follow-up, despite the progressive nature of the disorder. Health-related quality of life remained broadly unchanged in the other three patients and they did not continue the diet. Seizure frequency remained stable and blinded rating of their myoclonus showed improvement, albeit modest, in all patients.

**Conclusions:**

This observational, prospective study shows that some North Sea Progressive Myoclonus Epilepsy patients may benefit from the modified Atkins diet with sustained health-related quality of life improvement. Not all our patients continued on the diet, but nonetheless we show that the modified Atkins diet might be considered as a possible treatment in this devastating disorder.

## Background

North Sea Progressive Myoclonus Epilepsy (NSPME) is a rare but devastating disorder clinically characterized by progressive myoclonus, seizures, early-onset ataxia and areflexia. In the great majority of patients NSPME is caused by the same homozygous c.430G > T (p.Gly144Trp) mutation in the *GOSR2* gene, first reported in 2011 [1]. In 2013 Boissé Lomax and colleagues coined the term NSPME, as all the patients described came from countries bounding the North Sea [2].

The clinical picture of NSPME is dominated by spontaneous and action-induced myoclonic jerks and ataxia, which have a severe impact on daily functioning [3]. Most NSPME patients also have generalized tonic or tonic-clonic seizures, albeit that the seizures are relatively mild compared to the myoclonic jerks. Both myoclonic jerks and seizures can be treated with anti-epileptic drugs, but the benefits are disappointingly limited. Progressive myoclonic epilepsies, as a group, are not amenable to epilepsy surgery [4]. Vagus nerve stimulation can lead to seizure reduction, but does not help control the myoclonus [5]. This absence of an effective treatment for NSPME served as an impetus for exploring alternative treatment options.

The ketogenic diet (KD) has been proven to be effective in other drug-resistant epilepsy syndromes [6, 7], including those with myoclonic seizures [8, 9]. The modified Atkins diet (MAD) is a less restrictive variant of the classical KD and has shown similar benefits in seizure disorders [10]. The KD is a high-fat, low-carbohydrate diet. In contrast to the classical KD, the MAD does not restrict protein- or calorie-intake. The MAD is therefore easier to maintain, facilitating long-term compliance, especially in adolescents and young adults [11].

Our aim was to evaluate the efficacy of the MAD in patients with NSPME by measuring health-related quality of life (HRQL) as the primary outcome.

## Methods

### Participants

Four NSPME patients aged between 7 and 20 years, all with the known c.430G > T (p.Gly144Trp) mutation, participated in an observational, prospective, open-label study on the efficacy of the MAD in the University Medical Centre Groningen (UMCG) for 3 months (February to May 2013). They were 7, 12, 20 and 20 years old at the start of the trial. We offered six patients treatment with MAD, but two decided not to start with the diet. The study was performed according to the legal and ethical guidelines of the UMCG’s medical ethics committee.

### Modified Atkins diet

Pre-evaluation was made and dietary instructions given according to the recommendations of the international ketogenic diet study group during a 3-day hospital admission [12]. The MAD that was applied included a carbohydrate intake restriction, initially of 0.4 g per kilogram body weight, together with administration of the ketogenic formula: KetoCal 4:1 LQ®/ 12 ml per kilogram body weight. Fat and protein intake were unlimited. The diet was initiated stepwise at home over 7–10 days without fasting. When ketosis was adequately stabilized, carbohydrate intake was increased stepwise by 5 g per day as long as ketosis persisted. Patients were allowed to take extra carbohydrates, on the condition of taking 4 g extra fat for every gram carbohydrate. Blood ketones and glucose were assessed by twice-daily home monitoring for at least the first month. After the first month stable ketosis had been reached in all patients and blood ketones and glucose measurements were gradually reduced from twice a day to eventually only once a week. On indication patients or their caregivers performed additional sampling of glucose and ketones.

Our dieticians helped the caregivers to offer a wide selection of alternative products to the patients. For instance written dietary exchange lists were provided, and patients were allowed to compensate the carbohydrate intake with fat emulsion, so they could choose their preferred dietary products.

During the first 6 weeks, the patients visited our multidisciplinary outpatient clinic every 2 weeks. In addition, at least once a week the dieticians had an telephone or e-mail consultation with the patients or their caregivers, or more frequently when necessary (sometimes daily contact).

### Data collection

To evaluate the efficacy of the MAD we assessed several clinical parameters at baseline and after 3 months on the diet. HRQL was assessed by using the Dutch Generic Core Scale of the Paediatric Quality of Life Inventory (PedsQL) 4.0 [13]. Each patient completed the age-specific version of the PedsQL and, in addition, the parents of the two paediatric patients completed the PedsQL parent proxy report. The PedsQL questionnaire asks patients and their parents to indicate to what extent the patient encountered problems in the last few months before baseline in physical, emotional, social and school-related domains.

From 4 weeks before baseline up until the end of the study, the patients and/or their parents recorded the seizure frequency in a daily diary. They also recorded any adverse effects of the diet and their perception of the myoclonus severity on a 10-point scale. In addition, an EEG and blinded rating of the myoclonus severity were performed at baseline and after 3 months. For the myoclonus rating we performed a videotaped examination using a standardized protocol. These videos were scored by two independent raters (JG and RZ), blinded for the condition of the patient (baseline or on the diet); they used the Unified Myoclonus Rating Scale (UMRS) [14] and the Clinical Global Impressions Scale [15].

Furthermore, questionnaires were used to assess mood and behaviour. The self-rated version of the Inventory of Depressive Symptomatology (IDS) was used for the two adolescent patients [16]. Mood and behaviour in the two youngest patients were measured by a neuropsychologist consultation, with the Child Behaviour Check List completed by the parents, the Youth Self Report completed by the patients, and the Caregiver Teacher Report Form, completed by the school teacher. An occupational therapist used the Canadian Occupational Performance Measure (COPM) to identify and prioritize issues that restricted the patient’s performance in everyday living; this provided the basis for setting intervention goals [17]. After 3 months, changes in the patient’s self-perception of occupational performance were also evaluated with the COPM.

## Results

Patient characteristics are shown in Table 1. A detailed description of their clinical phenotypes has been reported elsewhere [3].

**Table 1:** Baseline characteristics of the patients

Patient	Sex	Age^a^	Motor function	Seizures	EEG	Medical treatment
1	M	12	Ambulant	Clonic seizures	GED, PCR	CLN, LEV, VPA
2	M	20	Ambulant + wheelchair	Tonic seizures	GED, PCR	CLN, ESM, LEV, VPA
3	M	20	Wheelchair	GTCS	GED, PCR	CLN, ESM, LEV, VPA
4	M	7	Ambulant	No	GED, PCR	None

^a^Age at start modified Atkins diet

*Abbreviations*: *CLN* Clonazepam, *EEG* Electroencephalography, *ESM* Ethosuximide, *GED* Frequent generalized epileptic discharges, *GTCS* Generalized tonic-clonic seizures, *LEV* Levetiracetam, *M* Male, *PCR* Photoconvulsive response, *VPA* Valproic acid

All patients completed a 3-months period on the MAD. The diet was well tolerated and none of the patients reported major side-effects. During this period there were no relevant changes in medication or in weight. The patients received 15, 19, 17 and 35 g of carbohydrates/day respectively, which was also based on bodyweight. Ketosis was reached within 2 weeks in all patients, but significant ketosis was only observed in the youngest patient (patient 4). He became ketotic after just five days on the diet and had average ketones of 4.3 mmol/L (range 2.5–6.6 mmol/L). The other child (patient 1) had average ketones of 2.3 mmol/L (range 1.3–3.7 mmol/L), while the two young adults had average ketones of 2.2 mmol/L (range 0.8–4.4 mmol/L) and 2.6 mmol/L (range 1.3–3.5 mmol/L), respectively. Stable ketosis was somewhat easier to achieve in the two youngest patients.

The results of the assessments at baseline and after 3 months on the diet are shown in Table 2. Patient 1 and his mother reported a 40% and 13% improvement in HRQL, respectively. The HRQL scores of patient 3 and 4 also showed improvement (5% and 14% respectively). Patient 2 and the parents of patient 4 reported a deterioration of the HRQL (19% and 39% respectively).

**Table 2:** Results of the assessments at baseline and at 3 months on the Modified Atkins Diet in four patients with North Sea Progressive Myoclonus Epilepsy

Patient	HRQL^a^				UMRS^b^				Effect on seizure frequency^c^	EEG change^c^	Changes in mood and behaviour^c^
	Baseline	MAD at 3 months	Change	MAD at 3 years	Baseline	MAD at 3 months	Change	MAD at 3 years			
1	pt. 46	pt. 27	impr. 19 pts	pt 47	71	64	impr. 7 pts	75	No change	No changes	n.d.
	par. 50	par. 43	impr. 7 pts	par. 37							
2	25	31	det. 6 pts	n.a.	72	58	impr. 14 pts	n.a.	No change	No changes	det. IDS 7 pts
3	58	55	impr. 3 pts	n.a.	87	85	impr. 2 pts	n.a.	No change	n.d.	det. IDS 8 pts
4	pt. 28	pt. 24	impr. 4 pts	n.a.	57	56	impr. 1 pts	n.a.	n.a	No changes	no relevant change
	par. 19	par. 31	det. 12 pts								

^a^scores were calculated by the sum of all scores divided by the number of answered items (maximum score 92). A lower score represents a better HRQL

^b^UMRS: scores represent the sum scores of section 2, 3 and items A-G of section 4 of the UMRS, calculated by using the UMRS score sheet [14]. A lower score represents less myoclonus; for patient 2, 3 and 4 the scores are the average scores of the two raters; for patient 1 the consensus scores of the two raters are shown in the table (because in the individual scores there was one outlier, so a consensus meeting was organised where the raters rescored all videos together)

^c^change between baseline assessment and 3 months assessment during the MAD

*Det*. Deterioration, *EEG* Electroencephalography, *HRQL* Health-related quality of life, *IDS* Inventory of depressive symptomatology, *impr*. improvement, *MAD* Modified Atkins diet, *n.a*. not applicable, *n.d*. no data, *par*. parents, *pt*. patient, *pts* points, *UMRS* Unified Myoclonus Rating Scale

Blinded rating of myoclonus (UMRS), showed small but positive changes in all patient scores (both in rest and in action). The most evident improvements in UMRS score were seen in patients 1 and 2 (aged 12 and 20 years). Seizure frequency remained stable in the three patients who suffered from seizures.

The EEGs of the four patients did not show a relevant decrease of epileptic discharges while on the MAD. Although patient 4 had presented no clinical seizures and only myoclonus and ataxia, his EEG did show epileptic activity and this did not change during the diet period. The results of the complementary assessments of mood, behaviour and occupational performance did not show relevant changes in the younger children, but the two adolescent patients reported negative effects. They felt more depressed due to the restricted diet, in particular they missed specific foods such as bread and potatoes in their daily diet. Their IDS-score declined by 7 and 8 points, respectively. Because the benefits did not match the efforts of maintaining the diet due to its restrictions, patients 2, 3 and 4 discontinued the MAD after a duration of 3, 3 and 5 months, respectively. The provided wide variation of alternative products and menus, and the intensive support from our dieticians could not prevent discontinuation. The parents of the youngest patient (patient 4, aged 7 years) said they would consider to restart the MAD in the future if their child’s symptoms would become more severe.

To date, patient 1 is still on the MAD and his HRQL and blinded rating of myoclonus were reassessed after 3 years on the diet. Compared to the baseline measures, he reported the same HRQL, while his parents reported a sustained improvement of 25%, despite the progressive nature of the disorder. In parallel, the UMRS scores at three years remained broadly unchanged compared to baseline. He showed an improved and sustained physical fitness on the diet, and he recently switched from a school for physically handicapped children to regular secondary education.

## Discussion and conclusion

Worldwide only 21 NSPME patients have been described [1–3]. In this observational prospective study, we evaluated the efficacy of the MAD in four NSPME patients, with HRQL as our primary outcome measure. In our study one of the four patients showed an improved HRQL on the diet. This 12-year old boy reported a significant (40%) improvement in his HRQL after 3 months on the MAD. He decided to continue on the diet because he felt healthier and less tired, experienced less jerking in the evening and less nocturnal shaking, and could participate more at school and in social events. After 3 years on the MAD, his HRQL has stabilized compared to his baseline, despite the progressive nature of the disease. The other three patients reported varied changes in their HRQL and UMRS while on the diet, but all decided to stop after 3 to 5 months because the benefits were perceived to be too limited compared to their dietary restrictions.

Ketosis in the youngest patient (#4) was excellent while he was on the MAD; compared to the other patients he had milder myoclonus and no clinical seizures, but a comparable HRQL to patient 2, for instance. In this respect it was interesting to observe that his parents thought the burden of the diet was more relevant than the reduction in his myoclonus: they reported a deterioration in the HRQL. However, the patient himself reported a considerable improvement in his HRQL questionnaires, which illustrates that the parents and patient experienced the diet’s burden differently, and this influenced their decision to continue the dietary treatment (Table 2).

The levels of plasma ketones of patient 1 were similar to those of patient 2 and 3. The benefit of the MAD observed in patient 1 and lack of benefit in patients 2 and 3 are therefore unlikely to be due to differences in the degree of ketosis achieved during the first 3 months.

Seizure frequency remained stable in all four patients while on the diet. Although epilepsy was not their main symptom, three of the four patients had generalized tonic, clonic or tonic-clonic seizures with a mean frequency of once a week. Neither the patients nor their parents reported a relevant decrease of seizure frequency while on MAD. EEG findings did not show a change in the epileptic discharges in any of the patients while on the MAD. The UMRS scores showed small, but positive, changes in all the patients on the diet, and the scores of patient 1 at 3 years remained broadly unchanged compared to his baseline, which is remarkable given the progressive nature of the disorder.

Reports on the effect of treatment with the KD in PME are scarce. The KD seems to be particularly effective in generalized forms of epilepsy, including epilepsies with myoclonus [9, 18]. The response rates in the randomized controlled trials of Neal et al. and Lambrechts et al. [6, 7] in children with refractory epilepsy were 38% and 50% respectively, with the percentage of patients who had >50% seizure reduction as the primary outcome measure. In our patients, not epilepsy but myoclonus was the major symptom, reported to interfere most with their activities of daily living. This makes it difficult to compare our results of the controlled KD trials. Despite our study showing improvement in only one out of four patients, for this single case MAD made a major and sustained difference to his HRQL and this was thus an excellent treatment result.

We chose HRQL as the main outcome measure and not seizure frequency or UMRS scores because we considered the sole use of impairment-focused measures to be too limited in scope to evaluate the overall effects of the diet effectively. It has been shown that disease severity rating scales might not always be suited to evaluating the overall effects of an intervention [19], and in small groups of patients it is difficult to detect minor differences on disease severity scales [20]. For these reasons we chose HRQL as our primary endpoint [19, 20]. Koy et al. supported this idea; they described how quality of life can improve significantly in children after deep brain stimulation for dystonia due to cerebral palsy, without any improvement shown on rating scales [21]. Moreover, HRQL likely includes all the different aspects of treatment sequelae in this very rare disorder, and importantly also takes into account the influence of the diet’s restrictions on the patient’s well-being. This is well illustrated by patient 2, in which an improvement of almost 20% was observed in blinded rating of his myoclonus, but the improvement was counteracted by a deterioration in his mood and a depressed state due to the diet.

There are several limitations to our study. First, we were only able to evaluate four patients and could not include a control group. However, given the rarity of NSPME and the number of patients reported worldwide with this disorder (*n* = 21), four patients is still a good size group to study. Second, the duration of follow-up of three of the four patients was relatively short because they decided to discontinue the diet.

In conclusion, this observational study shows that one out of four patients with NSPME had a favourable response to the MAD. This patient, who was 12 years old at the start of the study, has been on the diet for more than three years and has a stable HRQL, despite his progressive disorder. Therefore, the MAD might be considered in patients with NSPME, as it may improve or stabilize HRQL in this devastating disorder.

## Abbreviations

COPM: Canadian Occupational Performance Measure
EEG: Electroencephalogram
HRQL: Health-related quality of life
IDS: Inventory of Depressive Symptomatology
KD: Ketogenic diet
MAD: Modified Atkins diet
NSPME: North Sea Progressive Myoclonus Epilepsy
PedsQL: Paediatric Quality of Life Inventory
UMRS: Unified Myoclonus Rating Scale
